# The structure of forest vegetation on industrial landfills of different ages

**DOI:** 10.1002/ece3.10276

**Published:** 2023-07-07

**Authors:** Elena Gorozhanina, Dmitry Gura, Patryk Sitkiewicz, Tatyana Degtyarevskaya

**Affiliations:** ^1^ Department of Biology and General Genetic I.M. Sechenov First Moscow State Medical University Moscow Russian Federation; ^2^ Department of Cadastre and Geoengineering Kuban State Technological University Krasnodar Russian Federation; ^3^ Department of Geodesy Kuban State Agrarian University Krasnodar Russian Federation; ^4^ Faculty of Oceanography and Geography University of Gdańsk Gdańsk Poland

**Keywords:** dominant species, forest vegetation, Kuzbass quarry, Mosbass quarry, SDGs, Sokolovsky quarry

## Abstract

The purpose of the research is to describe plant community formation on the quarry surfaces to determine a path for optimal revegetation. To achieve the goal, the studies determined soil pH, the content of skeletal fraction, basal respiration, and performed the acidimetric assessment of CO_2_. The research program aimed to investigate the peculiarities of plant communities' formation in areas with different degrees of revitalization and investigate the influence of soil cover on plant associations. Results showed that the average basal soil respiration rate on the quarry was extremely low (about 0.3 mg CO_2_/g of soil/h). The CO_2_ content in the carbonate ranged from 0.07% to 0.7%, with the higher figures observed in older Kuzbass rather than Mosbass and Sokolovsky quarries. An analysis of soil samples from three quarries revealed four plant groups at sites associated with the prevalence of specific fractions, such as gravel, sand, silt, and stony soil. Given that Kuzbass is the oldest open‐pit mine, forest vegetation species dominate here in the surveyed areas (>40%), and this feature is typical of gravel soils. The dominant species present on the gravel substrate were downy birch (*Betula pubescens*), common hornbeam (*Carpinus betulus*), European oak (*Quercus robur*), Siberian spruce (*Picea obovata*), common juniper (*Juniperus communis*), Siberian larch (*Larix sibirica*), common pine (*Pinus*), and Siberian fir (*Abies sibirica*). Mosbass is also characterized by a diversity of similar species, though work on mineral mining there ended in 2009, more recently than in other sites. Stony and sandy soil fractions predominated in the Sokolovsky quarry, although other studied substrates were also present.

## Graphical abstract

The purpose of the research is to reveal the peculiarities of plant communities' formation on the territory of quarries, for the optimal revegetation in the respective territories. To achieve the goal, soil pH, the content of skeletal fraction, basal respiration, and acidimetric assessment of CO_2_ were determined. The research program was intended to investigate the peculiarities of plant communities' formation in areas with different degrees of revitalization and establish the peculiarities of soil cover influence on plant associations.

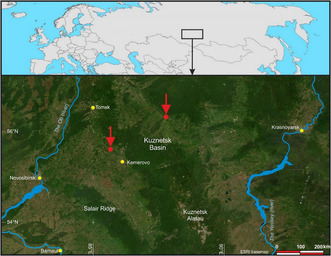

## 1 INTRODUCTION

For centuries, quarries have served as vital mining sites, reshaping the landscapes they occupy. This process has changed the landscape of many regions. Scientists who investigate the issue of quarries claim that places changed by natural disturbances represent groups of rare biodiversity (Talento et al., 2020). Many geological faults become ecological corridors and spaces of communication, shelter, and biological transit, thereby ensuring the survival of certain biomes (Roberts & Mattoo, 2018; Talento et al., 2020). Therefore, the territories modified due to mining activities can create other biodiversity repositories. With proper planning, abandoned open pits can help create new habitats for flora and wildlife (Lameed & Ayodele, 2010; Talento et al., 2020).

These abandoned lands have low levels of biomass productivity and quantity of resources, due to extreme abiotic environmental conditions (Roberts & Mattoo, 2018). However, extreme conditions are becoming increasingly rare as humans attempt to increase the productivity of the land. The early successional phase, dominated by rare representatives of the flora, deserves special attention among researchers (Hattermann et al., 2018). Given that quarries are an important part of the country's economic activity, they can increase biodiversity in certain regions, which is especially important in densely populated areas (Wang et al., 2018).

The mining industry of our planet annually extracts more than 100 billion tons of raw materials, and only about 7%–8% is used effectively. This leads to the radical restructuring of the geological structure of the Earth's surface to a depth of several 100 m. This change complicates or completely destroys the existence and functioning of organismal associations. The purpose of revegetation is to reproduce the productivity of the territories disturbed by the mining industry and return them to various types of use. This procedure involves the implementation of a complex of engineering, mining, meliorative, agricultural, and forestry works (Ramirez‐Cruz et al., 2019).

Over the centuries‐old history of mineral resource development, different‐age quarry and dump landscapes have formed. Currently, they are at different stages of development. Some of them are revegetated, but most are self‐regulating. Some of them can be classified as cultivated; they have long served as pastures and hayfields (Bartz & Kowarik, 2019).

Quarry and dump landscapes differ sharply from the surrounding zonal natural objects, as well as from other anthropogenically disturbed territories, due to a complex of specific plant growth conditions with contrasting ecological conditions and a special type of succession process. At the same time, human‐made ecotopes most fully reveal the adaptive capabilities of many plant species. Starting from the first stages of overgrowth, along with the group of weeds, species from the composition of the surrounding flora take an active part in it (Kowarik, 2019).

The biological reclamation of industrial dumps requires a comprehensive study. The study can make it possible to implement appropriate evidence‐based measures, in particular, the clarification of the natural overgrowth processes. An analysis of this process can determine possible ways of revegetation (Hölzel et al., 2016). Biological conservation of the surface of dumps is an urgent task, as their natural overgrowth is slow, and the formed vegetation groups are low‐productive (Skálová et al., 2015).

The natural vegetation of quarries and dumps depends on the following:

1. zonal and climatic conditions of the district,
2. the microclimate and physicochemical soil properties that determine the ecotopic selection of plants,
3. surrounding vegetation as a source of plant seed deposition on the barren surface of the dump.

In both natural and semi‐natural habitats, soil vegetation and related physicochemical processes are the main components of terrestrial ecosystems (Comerford, 2005). The consistent availability of nutrients for plant growth is an important factor that controls the nutrient cycle in soils for the primary production of plant communities (Spohn, 2020). However, when used in agriculture or forestry, individual parameters are often insufficient to monitor soil degradation and desertification or perform effective reclamation of soil substrate in post‐industrial territories. Factors affecting nutrient availability in soil are the first parameters to be considered when assessing soil quality and restoring substrate function while renovating ecosystem function (Skálová et al., 2015). Soil particulate fraction characteristics are the principal feedback indicators of the soil‐vegetation system (Hölzel et al., 2016). Some researchers have studied several soil‐vegetation relationships in more detail, including interactions between some tree species, desertification, and soil erosion (Wijitkosum, 2020).

In the scientific literature, there is a gap related to the knowledge of the relationship between soil physicochemical properties and vegetation functions that develop spontaneously in post‐industrial zones (Pourbabaei et al., 2020). These relations include the regulation of water infiltration and its availability for a specific plant community, as well as for the whole ecosystem (Fischer et al., 2014). Most studies examine the role of particulate fraction and soil properties in post‐industrial sites in the rehabilitation process (Fischer et al., 2014).

Several studies have analyzed the effect of the soil particulate fraction on the stability of the soil vegetation system due to regeneration processes (Halecki & Klatka, 2021). The significance of the fine fraction in soil processes has been demonstrated in numerous studies (Hölzel et al., 2016; Kowarik, 2019; Wijitkosum, 2020). Compared with soil in natural and semi‐natural ecosystems, the relationship between soil particulate fractions, water, nutrient availability, and spontaneous feedback of vegetation in post‐industrial sites is significantly more complex. This issue requires an analysis regarding practical restoration purposes.

The process of quarry regeneration is a relatively slow process, which can take years (Krüger et al., 2017). Limited nutrients and the physical condition of the soil are the main factors affecting the development of flora in areas of abandoned quarries (Fischer et al., 2014). A restored community must be capable of developing natural processes without human intervention (Hong et al., 2019). In addition, a recovered ecosystem must resist stress and include a set of native species (Lugo, 2020). Plant species should grow in rock conditions, in soils with low nutrients and water content, in soils with very high or conversely low pH values, or in areas located on steep slopes. Due to soil disturbances in quarries, the succession of plants is slow, requiring up to several centuries, especially for forest vegetation. Therefore, it is necessary to evaluate all possible approaches to the restoration of abandoned quarries, taking into account changes in the ecological role and biodiversity of fauna.

Forest plantations are recognized as a powerful soil‐forming factor that significantly affects the morphological structure, physicochemical and biological properties. When creating forest plantations, the focus is on the selection of an assortment of tree species that can increase the fertility of soil mixtures (Halecki & Klatka, 2021; Kowarik, 2019; Wijitkosum, 2020).

The acceleration of soil formation in quarry dumps can be possible due to the creation of tree and shrub plantations, as well as grass vegetation. These structures can ensure the optimal water regime in soils and a balanced ratio of nutrients (Garófano‐Gómez et al., 2017; Halecki & Klatka, 2021; Hong et al., 2019; Krüger et al., 2017; Lugo, 2020). The negative influence of soil erosion on forest vegetation occurs in conditions of extreme pH values.

Survival and growth of tree species, as evidenced by studies in this field (Beretta et al., 2014; Garófano‐Gómez et al., 2017; Kim & Kupfer, 2016), mainly depend on the skeletal fraction content of soil and its acidity. The most acceptable pH values for most species recommended for planting on disturbed lands are in the range of 4.0–8.0. The optimal pH values for coniferous species are 4.5–6.0, and for deciduous species are 6.0–7.5.

Carabassa et al. (2018) studied the effect of the soil's physicochemical properties on the passage of successional processes. They found that the added silt increases the amount of organic matter, consequently, successional plant species colonize renewal zones. Sewage sludge can be used as soil amendments (Carabassa et al., 2018). However, these additives often contain heavy metals that negatively affect the vegetation cover. In limestone quarries, ecological renewal of the biotope can take place within a few years after the use of backfill. Gentili et al. (2020) determined that soil granularity plays a key role in regeneration. The authors showed a linear dependence of the grass cover on the age of the renewable area of 3 × 3 m and the amount of organic and mineral matter. In addition, species diversity is affected by the stony composition of the soil and the sand content (Gentili et al., 2020).

Succession is crucial, especially for the renewal of grass cover, but its role is even more considerable regarding forest vegetation. Therefore, it is necessary to study the influence of soils on the renewal of woody cover during natural renewal processes. This study employed a more general approach to evaluate the relationship between the size of soil particles in coal mine tailings and the diversity of forest vegetation that developed during spontaneous succession. The evaluation of many samples allowed classifying the soil of vegetated areas according to the predominant proportion of particles (stone, gravel, sand, silt) on the research site.

Given that open‐pit mines have significant differences, the purpose of this study was to investigate the vegetation and soil characteristics of quarries of different ages on the territory of the Russian Federation and the Republic of Kazakhstan. This procedure included the following tasks:

1. selecting open‐pit landfills of different ages and climatic conditions to investigate the peculiarities of their soil formation,
2. identifying the main physical and chemical properties of the soil,
3. analyzing the baseline diversity of forest vegetation species for future comparison.

## 2 MATERIALS AND METHODS

### 2.1 Study regions

**FIGURE 1:**
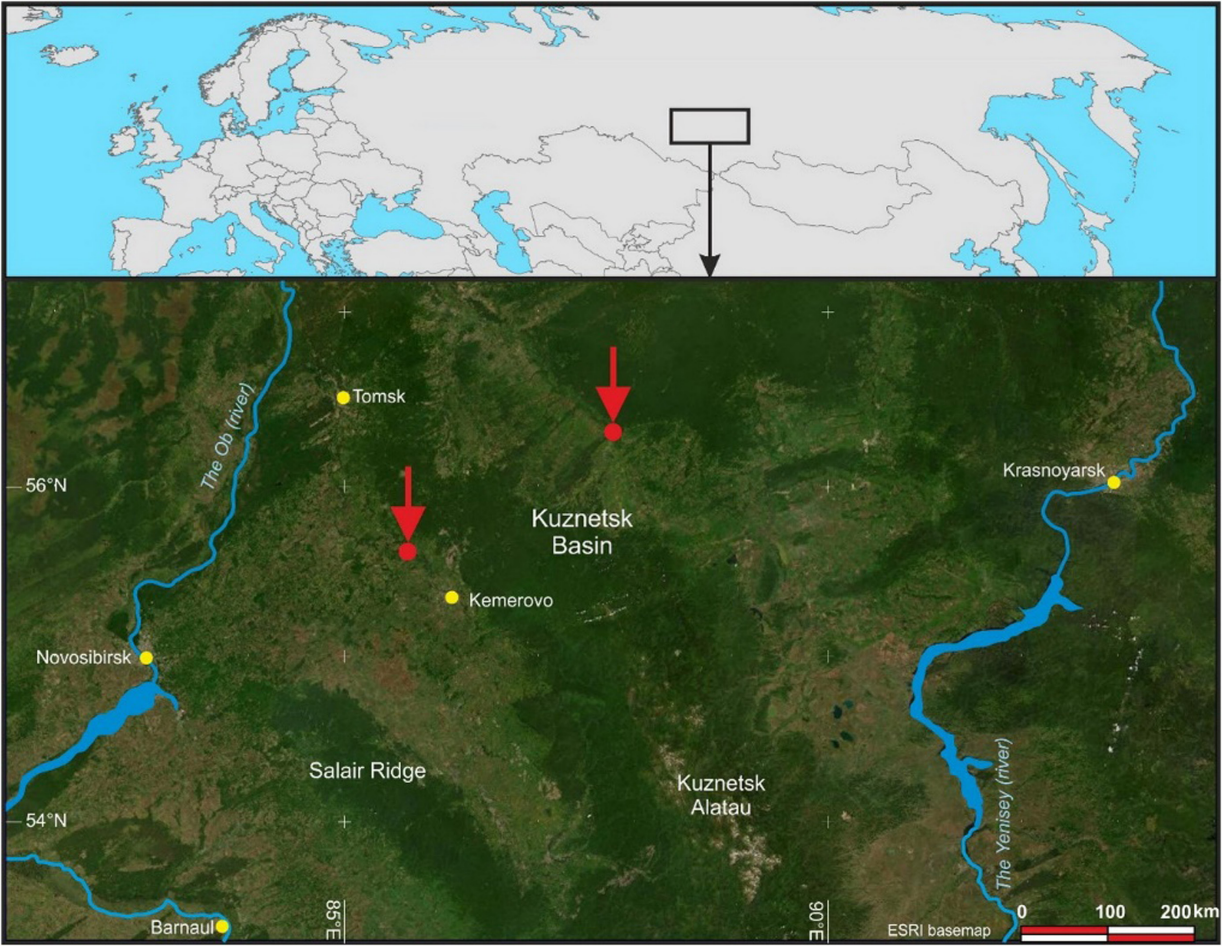
Soil sampling points on the territory of Kuzbass in 2001–2021.

The studies took place on the territory of the Russian Federation in the Kuznetsk coal basin (Kuzbass) (55°21′16″N 86°05′19″E) (Figure 1) in the spring–summer season of 2001–2021. The first data on this site date back to 1721. The climate of the region is strongly continental with frequent and sharp fluctuations in air temperature, amount of precipitation, and the intensity of solar radiation. These features are due to the region's geographical location in the south of Western Siberia.

The primary type of vegetation is southern taiga with conifer forests. However, the high incidence of natural and anthropogenic disturbance has led to the formation of broad‐leaved forests. Ecosystems are also characterized by sub‐boreal flora due to increased carbonate content in materials of local origin.

**FIGURE 2:**
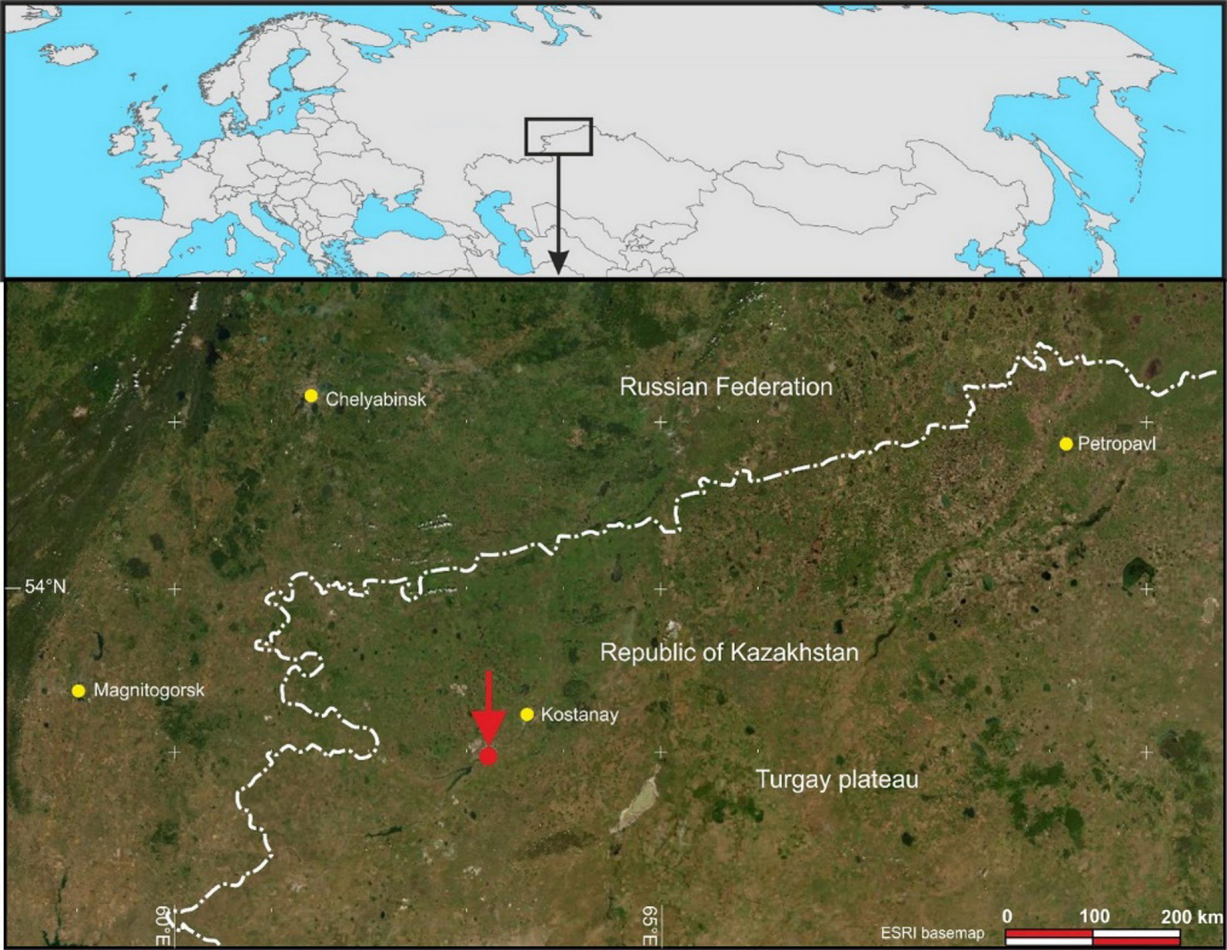
Soil sampling points on the territory of Sokolovsky quarry (Kazakhstan) in 2001–2021.

The research conducted during 2001–2021 also included observations at Sokolovsky quarry (Republic of Kazakhstan), which lies 45 km southeast of Kostanay City (Figure 2). It was discovered in 1948 and explored during 1949–1955.

The Sokolovsky quarry and its dumps are located within the boundaries of the Kostanay physical‐geographic province. The territory of latter is a sloping undulating plain that gradually descends to the northeast, with absolute elevations of 150–200 m. It belongs to the subzone of arid steppes on southern loamy, sandy, low‐humus, and often saline chernozems. Its overgrowth depends on the edaphic properties of soil mixtures and mesorelief forms. The climate is strongly continental, with a pronounced four seasonal alternation. The mean yearly temperature is 2.8°C. The ground cover here is primarily represented by dark brown and solonetz soils.

**FIGURE 3:**
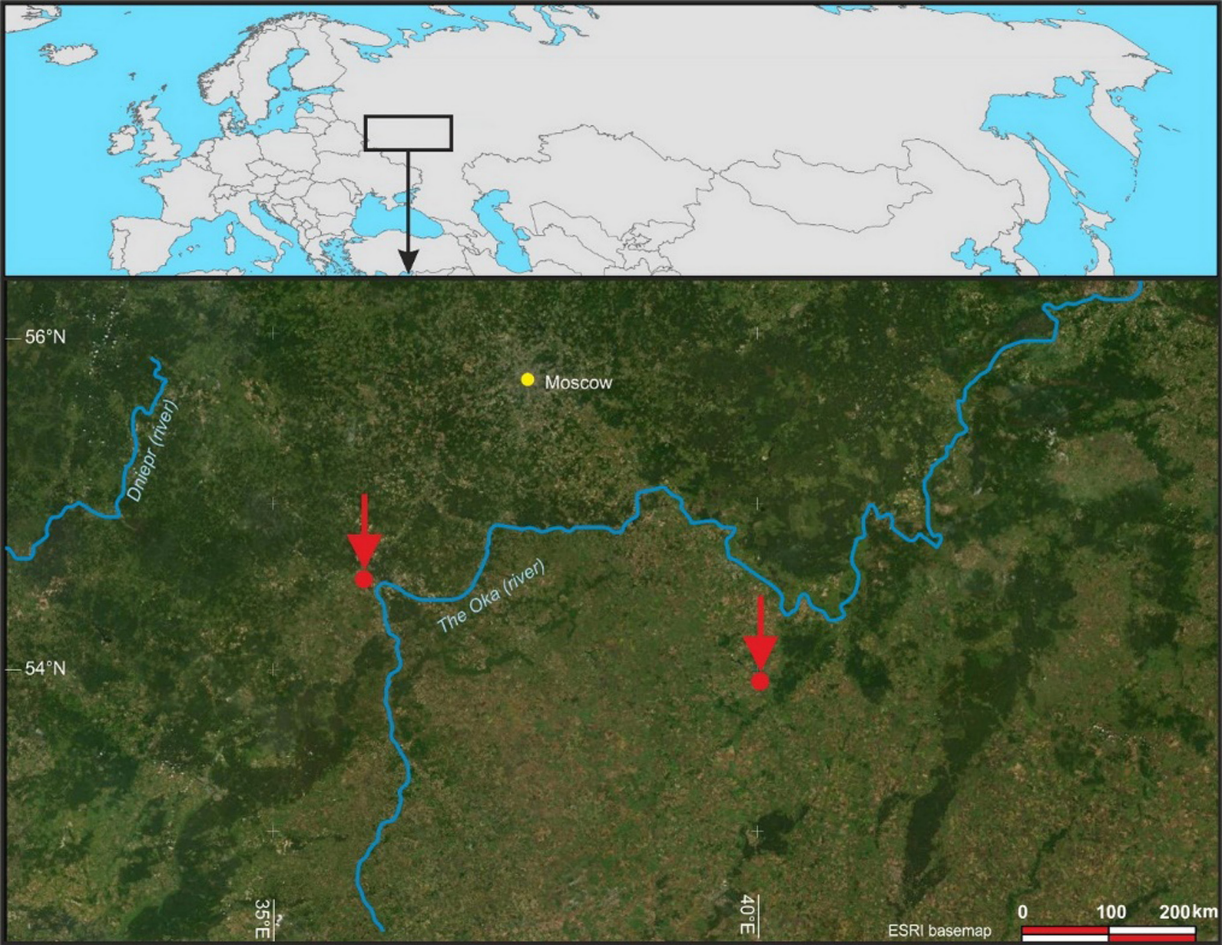
Soil sampling points on the Moscow Region Coal Basin (Mosbass) during 2001–2021.

In addition to the previous locations, this research was also carried out on the territory of the Moscow Region Coal Basin (Mosbass, Russia) (53°57′N 37°42′E), where coal was mined from 1855 to 2009 (Figure 3).

This area has a moderately continental climate, characterized by relatively warm summers and moderately cold winters. The landscape is dominated by forest‐steppe and steppe. Soils are primarily represented by moderately thick chernozems.

### 2.2 Sample selection

The experiment consisted of the following stages:

1. Establishing experimental plots;
2. Soil sampling and analysis;
3. Study of forest cover.

To determine the main forest inventory assessment indicators of the experimental forest stands, experimental trial areas were established. The procedure complied with the methodology of conducting field research using the field geographic information system Field‐Map (IFER‐Monitoring and Mapping Solutions. S.r.o., www.field‐mapping.com). In field conditions, this methodology allows researchers to combine the formation of attributive and cartographic information about forest objects in a single technological process.

Given that small‐scale topographic differences strongly influence the soil and vegetation succession process (Hong et al., 2019), the research involved sites with various topographic features (Figures 1, 2, 3). Kuzbass is located in a shallow basin between the mountain ranges of the Kuznetsk Alatau, Mosbass is on the Central Russian Upland, and Sokolovsky is in the southeastern part of the West Siberian Plain.

On the Kuzbass and Mosbass, soil material was sampled from two locations in each quarry with subsequent averaging of the results. In the Sokolovsky quarry, the samples were collected from one place. There were 50 search points selected at each location. The research also included a survey of the flora in the quarries. On each ecotype, areas of 10 × 10 m were created. All types of forest vegetation (mature trees) were identified at each site.

An analysis of soil morphology and characteristics took place under laboratory conditions. Soil samples were ground and passed through a 1.5 mm sieve. The soil sample analysis aimed to identify the following parameters: pH in aqueous‐saline slurry (1: 3), the skeletal fraction content, the size distribution of the soil using the pipette method with the pyrophosphate peptization micro aggregates (Lugo, 2020), basal respiration soil, acidimetric assessment of CO_2_ in carbonates (Beretta et al., 2014; Dettori & Donadio, 2020; Kim & Kupfer, 2016).

### 2.3 Statistical analysis

MANOVA (multivariate analysis of variance) was used to process the results. The analysis employed Microsoft Excel and Statistica 10 software packages. In the study, MANOVA was performed on a standardized data matrix to alternately test the qualitative variables available in the database. This program allowed the authors to calculate the parameters of species diversity. In addition, the study used a correlation analysis to relate the vegetation species composition with the size of soil particles and characterize the relationship between the floristic composition of forest vegetation areas and the measured physicochemical soil characteristics (Smith, 2018). The set threshold for statistical significance was *p* ≤ .05.

### 2.4 Data use

Since species diversity may be exaggerated by the presence of rare species, the calculations employed Simpson and Shannon–Wiener (L) diversity indices. These are weighted expressions of species richness and abundance of each species in the population (Beretta et al., 2014). Simpson's index of diversity is calculated as follows:

$$L=1-\frac{\sum n\left(n-1\right)}{N\left(N-1\right)}$$

∑ = sum; *n* = the number of individuals for each species; *N* = the total number of individuals for all species in the population.

The Shannon–Wiener diversity index (H) is calculated as follows:

$$H=-\sum \mathrm{Pi}\left(\text{In}\mathrm{Pi}\right)$$

*Pi* = share of the number of individuals (abundance) of the *i*‐th species, ln = natural logarithm.

Species evenness, which is the relative number of species per unit area, was estimated as the Pielour evenness index (J):

J=H/S.

H and S are as defined above.

The authors applied these methods according to their characteristics and the effectiveness of their implementation. Namely, Simpson's dominance index indicates the dominance of certain types of grouping. It also describes the probability of the relationship of any two individuals, randomly selected from an indeterminately large grouping to different species. A relatively popular Shannon–Wiener index is sufficiently informative. It reflects the complexity of the group structure, without being an index of species richness, it reveals a strong dependence on the number of species. Therefore, it is suitable for assessing species diversity. An increase in the Pielour index indicates the evenness of the grouping structure. The quantitative characteristic of species diversity tends to reach the maximum possible value. The block diagram of the methodology presented in the study aims to analyze development conditions, including soil conditions, the adaptation of green plantings to the forest layout, and the development of a greening/revegetation plan.

## 3 RESULTS

To compare the distribution of forest vegetation in quarries, the research took place not only in Kuzbass but also on the territory of other quarries characterized by different geographic conditions and ages. A variety of landscapes and ecotopes distinguishes the quarry of the Kuznetsk coal basin. A wide variety of soil surfaces was found within the quarry, confirming a multidimensional pattern of ecosystem development. The analysis of soil physicochemical parameters showed a relatively high value of the soil transformation factor. The index of soil acidity in the surface regions decreased over the years to 5.0 due to the accumulation of organic matter. There was a similar trend in the territory of the youngest quarries—Mosbass and Sokolovsky, although the pH values increased.

**FIGURE 4:**
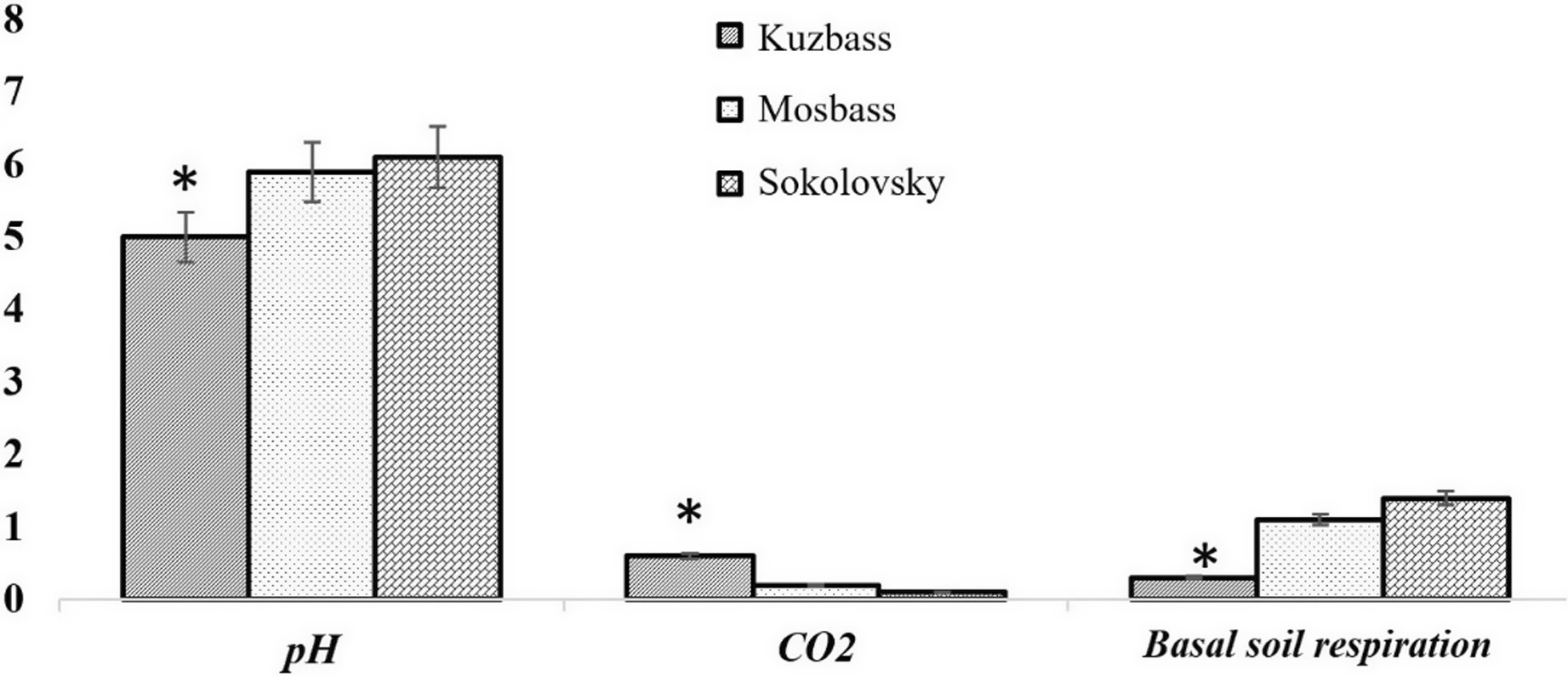
Physicochemical properties of soil samples from Kuzbass, Mosbass, and Sokolovsky quarries: pH, CO_2_ (%), and basal soil respiration indices (mg, CO_2_/g soil/h). *The values are statistically significant at *p* ≤ .05.

Surprisingly, the lowest pH values were in the relatively high arable soil. All areas were characterized by a highly heterogeneous distribution of fractions within the profile. In all regions, a large amount of coarse skeletal material had a comparatively low content of fine soil. Rockiness variations (from 8.0% in regenerated areas to 65% on a rising background) indicate heterogeneity of conditions in the quarry. This indicator largely depends on the recovery technology applied. The recorded high levels of CO_2_ are a negative consequence of mining, particularly in the case of carbonate rocks. Carbon dioxide emissions can be estimated by observing basal soil respiration (Figure 4). During vegetation growth, CO_2_ is deposited, and the rate of this reaction can be estimated by examining the amount of organic carbon in the soil composition. Basal soil respiration was low (0.3 mg CO_2_/g soil/h). The principal sources of CO_2_ emissions in the atmosphere are altered carbonates. During the research, the content of CO_2_ in carbonate ranged from 0.07% to 0.7%. The highest indices were in the territory of the older Kuzbass and not in the younger mines of Mosbass and Sokolovsky quarries.

**FIGURE 5:**
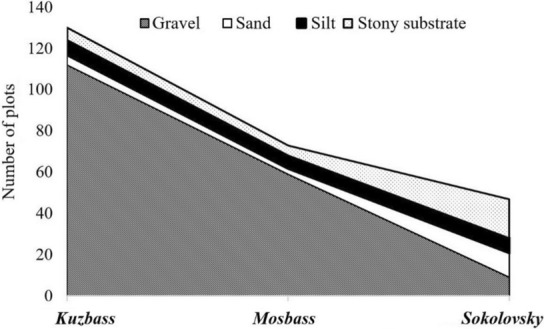
Distribution of soil type in the studied areas of Kuzbass, Mosbass, and Sokolovsky quarries.

The analysis of soil samples from three quarries revealed three species groups in areas associated with the dominance of certain fractions, such as gravel, sand, and silt. The fourth group consisted of areas with predominant species closely related to the stone fraction. Within the study, most of the forest vegetation areas (180) were on a substrate with a high proportion of gravel (Figure 5). A total of 98 dominant species were found on these sites, occurring with an average frequency of 15.67% on this type of substrate.

**FIGURE 6:**
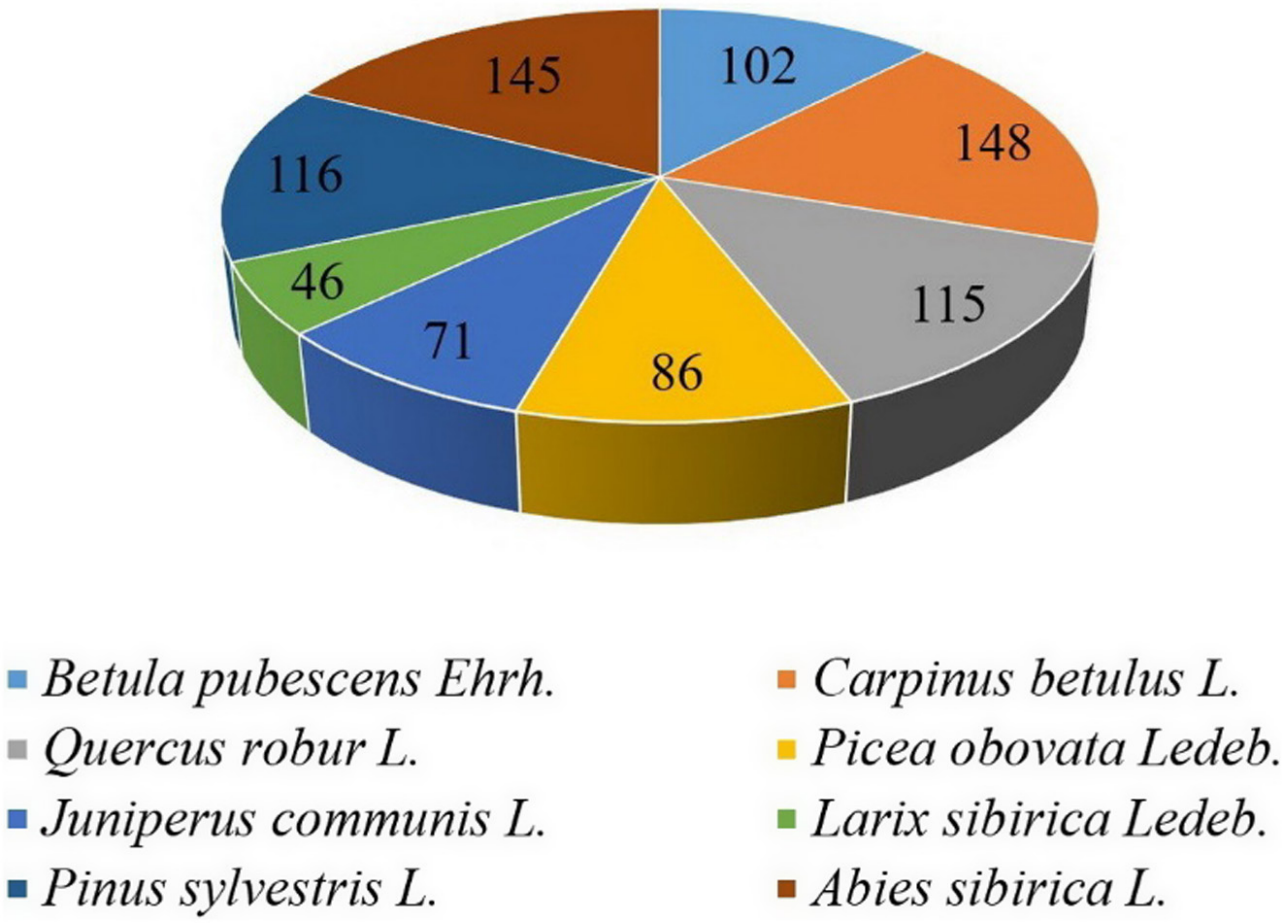
The number of plots with dominant species of forest vegetation on the gravel substrate of the studied landfills of Kuzbass, Mosbass, and Sokolovsky quarries.

The dominant species found on this type of substrate were Downy Birch (*Betula pubescens Ehrh*.), Common Hornbeam (*Carpinus betulus L*.), European Oak (*Quercus robur L*.), Siberian Spruce (*Picea obovata Ledeb*.), Common Juniper (*Juniperus communis L*.), Siberian Larch (*Larix sibirica Ledeb*.), Scotch Pine (*Pinus sylvestris L*.), and Siberian Fir (*Abies sibirica L*.) (Figure 6).

The vegetation was the lowest in areas with chalky soils since they had low species diversity. Among this type of substrate, there were nine dominant species with an average frequency of 5.14%. The vegetation of these regions was most often dominated by the Common Willow (*Salix acutifolia Willd.*). A similar frequency (5.65%) occurred for vegetation areas on sand substrates. Here, downy birch (*B. pubescens*) (18) and Common Barberry (*Berberis vulgaris L.*) (13) prevailed. Downy Birch (*B. pubescens*) (23), Stone Oak (*Quercus ilex L.*) (25), and Norway Willow (*S. acutifolia*) (18) predominated on the stone substrate from 30 study points.

Table 1 shows the parameters of species diversity according to the specified indices.

**TABLE 1:** Species diversity parameters of Kuzbass (S1), Mosbass (S2), and Sokolovsky quarries (S3).

Species diversity parameters	S1	S2	S3
Simpson's index	0.863245	0.789652	0.679163
Shannon index	2.023654	1.986321	1.821151
Pielour evenness index	0.896541	0.596972	0.493254

Given that Kuzbass is the oldest open‐pit mine, here, forest vegetation types prevail in the studied areas, which is characteristic of gravel soils. Mosbass is characterized by various similar species, although work on mining minerals ended not that long ago (in 2009). As for the Sokolovsky quarry, the stony and sandy soil fractions predominate, although there were other studied substrates, in particular silt (Figure 5). The indices of diversity in Kuzbass were correspondingly higher than those in Mosbass and Sokolovsky quarries. This fact is also associated with the structural and aggregate composition of the soil, in particular with lower silt content.

## 4 DISCUSSION

Soil substrate structure and lack of water and nitrogen have long been considered as probable reasons for difficulties in creating plant cover in highly industrialized regions (Dettori & Donadio, 2020; Smith, 2018). There is a common opinion that abiotic factors are the driving forces that shape the structure of the vegetation of many terrestrial ecosystems. The latter include those created in post‐industrial and urban areas (Heip et al., 1998).

The principle of landscape formation and development involves the organization of space taking into account the historical conditions of formation (part of the industrial region) and the dynamic development of the territory (post‐technological territory) with the preservation and restoration of disturbed territories. The main features of disturbed territories are landfills, dumps, and quarries. Active measures for the biological reclamation of these dumps are the only real possibility to return the lost fertility and economic value to the lands occupied by the dumps in a relatively short time.

It was found that the texture of the soil substrate was one of the most critical factors determining the diversity of vegetation in the landfills of Kuzbass, Mosbass, and Sokolovsky quarries. The observed variety of spontaneously developing vegetation in the landfills of coal mines was revealed due to the physiognomic characteristics of the substrate areas. It is reflected in several abiotic factors that create a mosaic of different habitats. Moreover, some species registered in this study (such as *B. vulgaris* and *Q. ilex*) were considered rare for the industrial regions of the Russian Federation (Fields, 2004).

The composition of the bedrock, which is the basis of coal mine landfills with plants, depends on the weathering process of waste lying in these landfills. In turn, they largely depend on the type of stored rock and its physicochemical properties (Qiu et al., 2018; Van Puijenbroek et al., 2019). We have found that the predominant fractions of particles were gravel, sand, and silt. They had differences in the quantitative and qualitative composition of forest vegetation. The continuous process of alteration, the partial segregation of stone materials, and the development of plant succession constantly modify the proportion of certain soil particles in the soil substrate (Czerepanov, 1995). Due to the substrate's complex petrographic and granulometric structure in these landfills, soil processes are slow. Besides, the thickness of the hummus/organic layer can vary from a few millimeters (in the initial phase of succession) to several centimeters (in the later stages of succession). In shallow soil substrate layers, the size of soil substrate particles changes, along with the contents of some chemical compounds (Nguemezi et al., 2020).

The creation of new tree stands is well‐known in restoration ecology (Castle et al., 2016; Lima et al., 2018; Poirier et al., 2020; Schoonover & Crim, 2015). This method of tree planting and associated maintenance require the allocation of significant resources. Nevertheless, they do not always produce satisfactory results, for example, when continued maintenance is required (Liu et al., 2020; Poirier et al., 2020). It implies the revegetation of industrial quarries that evolve through natural processes (e.g., colonization and succession).

When succession occurs, plants, which are typical for the surrounding area, are those that appear in quarries most often. Thus, *Quercus pubescens*, *Fraxinus ornus*, *Acer campestre*, as well as some exotic species, such as *Pinus nigra*, *Acinos arvensis*, and *Malva ignoreta*, were found in limestone quarries. They could appear as a result of changes in soil characteristics (Boscutti et al., 2017). At the same time, in China, the study of the vegetative processes of coal mine renewal revealed the presence of *Ulmus pumila*, *Ailanthus altissima*, *P. tabuliformis*, and *P. simonii*. This may be due to the fact that the acidity of soils after coal mining is significantly lower than that of limestone (Yuan et al., 2018). In our study, the dominant species were *B. pubescens*, *Q. ilex*, and *S. acutifolia*, which are common in the territories adjacent to the quarries.

The activity of quarries is the complete removal of vegetation, which serves as a habitat and niche for wild animals. Only some serial or successional species, which are more adaptable, can take advantage of temporary and unstable situations. However, the complex manipulation of the environment in a quarrying project, directly or indirectly (habitat modification), will lead to serious consequences where rare and endangered species become maladaptive. In this case, more adaptive species, whose numbers are smaller, are preserved due to pollution (Lameed & Ayodele, 2010). According to several authors, the protection of the quarry area creates a chance to increase the recreational area in order to combine industry and preserve the geological heritage. At the same time, it can contribute to the economic and social development of local communities. If managed well, this strategy can bring economic and social benefits to the region (Lugo, 2020; Talento et al., 2020).

Overcoming land degradation through integrated landscape management should become a priority in the policies of departments related to ecology and revegetation. The results of the present research have shown that the increase in the amount of organic matter in the soil composition is the main process of soil formation. It leads to the formation of layers with the maximum amount of organic matter in the accumulative ecotype with optimal substrate moisture regime and physical parameters. These processes form horizons with the maximum thickness and the highest organic carbon content in the accumulative ecotype with optimal substrate moisture conditions and physical parameters. The differences between the surface and underground horizons decreased with the overgrowth time. This change may be due to the high intensity of organic matter accumulation in the soil (Egli et al., 2018). Along with an increase in organic matter accumulation, the acidification and leaching of carbonates took place rather quickly. That was especially noticeable in the upper layer of the Kuzbass soil horizons rather than in other quarries. In some forests, such as small‐leaved forests, soil formation processes are more rapid than in eluvial ecotypes (Czerepanov, 1995) An exception is a substrate with high density and excessively compacted bedrock; these properties impede the development of plant communities. The diversity of species from industrial landfills is determined by changes over time and different habitats. Over time, the values of these indicators increase and reach their maximum values in the oldest landfills (Dettori & Donadio, 2020; Gorozhanina et al., 2022). The results in this work show that soil substrate structure influences species richness and the diversity of developing vegetation species. There were significant differences in species richness and diversity in areas with a predominance of the stony fraction in the soil substrate compared to the silty fractions. In general, these indicators were at their highest values for stony and gravel substrates. Difficult habitat conditions on muddy substrates (low potassium content and irregular soil structure) have probably resulted in fewer species colonizing these habitats (the youngest Sokolovsky quarries) during vegetation development (Halecki & Klatka, 2021; Poirier et al., 2020).

The process of revegetation in quarries with different soil compositions has already been well studied. Most of the studies are devoted to various aspects of vegetation cover succession (Schoonover & Crim, 2015). Some studies have analyzed the soil formation processes in sufficient detail (Lugo, 2020). Thus, previous studies have long considered the structure of the soil substrate and the lack of water and nitrogen as probable causes of difficulties in creating plant cover on the territories of industrial facilities (Lugo, 2020; Van Puijenbroek et al., 2019). This conclusion was confirmed by the present research. Abiotic factors are generally believed to be the driving forces that shape the vegetation structure of many terrestrial ecosystems, including those created in post‐industrial and urban areas (Qiu et al., 2018). This fact adds value to the research performed. However, the present work lacks comprehensive observations on soil and forest cover restoration and the relationship between these features.

Natural and semi‐natural habitats, soil, vegetation, and their associated physicochemical processes require careful management to protect and proactively manage the long‐term valuable landscape and cultural assets that society aims to preserve.

## 5 CONCLUSIONS

The paper describes the features of soils and forest vegetation at quarry sites of different ages and climatic conditions, namely Kuzbass (Russia), Mosbass (Russia), and Sokolovsky quarry (Kazakhstan), which were studied from 2001 to 2021. Mesomorphological and chemical studies have shown relatively high conversion coefficients of the soil substrate in Kuzbass. As the soil ages, the amount of organic matter increases, leading to a decrease in soil pH to 5.0. A similar trend was observed in the younger Mosbass and Sokolovsky quarries, although the pH values were higher.

The main sources of CO_2_ emissions into the atmosphere were modified carbonates. The CO_2_ content in the carbonate ranged from 0.07% to 0.7%, with the highest rates in the older Kuzbass than in the younger Mosbass and Sokolovsky quarries. The analysis of soil samples from three quarries revealed four groups of species in areas associated with the predominance of such fractions as gravel, sand, and silt, with gravel being the most common type of soil. The results of the studies showed that the structure of the soil substrate affects the diversity of vegetation, while significant differences in species diversity are in areas with a predominance of the stony fraction in the soil substrate compared to the silty fraction. The research results have practical value in terms of their use for the creation of species biodiversity for growing in existing conditions on the territory of industrial facilities. Research recommendations can be useful when creating reclamation programs aimed at restoring the soil and plant environment after a technogenic disturbance.

## AUTHOR CONTRIBUTIONS

**Elena Gorozhanina:** Methodology (equal); resources (equal); supervision (equal); validation (equal); writing – original draft (equal). **Dmitry Gura:** Conceptualization (equal); funding acquisition (equal); software (equal); validation (equal); writing – review and editing (equal). **Patryk Sitkiewicz:** Data curation (equal); formal analysis (equal); validation (equal); visualization (equal); writing – original draft (equal). **Tatyana Degtyarevskaya:** Conceptualization (equal); investigation (equal); project administration (equal); validation (equal); writing – review and editing (equal).

## FUNDING INFORMATION

This research did not receive any specific grant from funding agencies in the public, commercial, or not‐for‐profit sectors.

## CONFLICT OF INTEREST STATEMENT

The authors have no competing interests to declare.

## Data Availability

All additional data are included in Google server on the authors mail. The access link—https://drive.google.com/drive/u/2/folders/1OzGYlUINfdF‐Fqek9d6HquLHGzymt8kx.
